# *ITS2 *secondary structure improves phylogeny estimation in a radiation of blue butterflies of the subgenus *Agrodiaetus *(Lepidoptera: Lycaenidae: *Polyommatus *)

**DOI:** 10.1186/1471-2148-9-300

**Published:** 2009-12-26

**Authors:** Martin Wiemers, Alexander Keller, Matthias Wolf

**Affiliations:** 1 Department of Animal Biodiversity, Faculty of Life Sciences, University of Vienna, Rennweg 14, 1030 Wien, Austria; 2 Department of Bioinformatics, Biocenter, University of Würzburg, Am Hubland, 97074 Würzburg, Germany

## Abstract

**Background:**

Current molecular phylogenetic studies of Lepidoptera and most other arthropods are predominantly based on mitochondrial genes and a limited number of nuclear genes. The nuclear genes, however, generally do not provide sufficient information for young radiations. *ITS2 *, which has proven to be an excellent nuclear marker for similarly aged radiations in other organisms like fungi and plants, is only rarely used for phylogeny estimation in arthropods, although universal primers exist. This is partly due to difficulties in the alignment of *ITS2 *sequences in more distant taxa. The present study uses *ITS2 *secondary structure information to elucidate the phylogeny of a species-rich young radiation of arthropods, the butterfly subgenus *Agrodiaetus*. One aim is to evaluate the efficiency of *ITS2 *to resolve the phylogeny of the subgenus in comparison with *COI *, the most important mitochondrial marker in arthropods. Furthermore, we assess the use of compensatory base changes in *ITS2 *for the delimitation of species and discuss the prospects of *ITS2 *as a nuclear marker for barcoding studies.

**Results:**

In the butterfly family Lycaenidae, *ITS2 *secondary structure enabled us to successfully align sequences of different subtribes in Polyommatini and produce a Profile Neighbour Joining tree of this tribe, the resolution of which is comparable to phylogenetic trees obtained with *COI+COII *. The subgenus *Agrodiaetus *comprises 6 major clades which are in agreement with *COI *analyses. A dispersal-vicariance analysis (DIVA) traced the origin of most *Agrodiaetus *clades to separate biogeographical areas in the region encompassing Eastern Anatolia, Transcaucasia and Iran.

**Conclusions:**

With the inclusion of secondary structure information, *ITS2 *appears to be a suitable nuclear marker to infer the phylogeny of young radiations, as well as more distantly related genera within a diverse arthropod family. Its phylogenetic signal is comparable to the mitochondrial marker *COI *. Compensatory base changes are very rare within Polyommatini and cannot be used for species delimitation. The implementation of secondary structure information into character-based phylogenetic methods is suggested to further improve the versatility of this marker in phylogenetic studies.

## Background

Molecular phylogenetic studies aim to reconstruct species trees, e.g. to infer the evolution of morphological characters or life history traits. While in the early days of genetic analyses, the data sets were often confined to single gene fragments, it is now generally acknowledged that analyses should include several genes [1-3]. The use of multiple genes not only provides a greater resolution over different time scales but yields a more accurate estimate of the species tree which may not correspond to a single gene tree, especially in radiations of closely related species [4,5]. Unfortunately, the number of genes which are routinely used for phylogenetic analysis, especially in species rich arthropod assemblages, have remained limited [6]. In the mitochondrial genome, the cytochrome c oxidase subunit I (*COI *) has become the most commonly used marker in molecular phylogenetic studies of arthropods, in part due to it being the focal genetic marker for DNA barcoding studies [7]. This marker is now routinely supplemented by the nuclear marker elongation factor 1 alpha (*ef1α *) and sometimes wingless (*wg *) [3,6]. These nuclear markers, however, continue to be of limited use in resolving the phylogeny of young radiations because of their slow evolutionary rate. Recently, novel nuclear genes have been tested in species of Lepidoptera, four of which (*Tektin, CAD, DDC, IDH *) appear promising for such radiations [6,8]. However, experience with these remains limited or lacking.

The internal transcribed spacer 2 (*ITS2 *), which separates the nuclear ribosomal genes *5.8S *and *28S *, constitutes a rapidly evolving nuclear DNA fragment and has proved very useful when inferring phylogenetic relationships of closely related species in groups of organisms such as plants and fungi [9]. The highly conserved flanking regions can be used as an anchor for universal primers. However, *ITS2 *studies on the phylogeny of metazoans are relatively rare. In arthropods, only 11,927 *ITS2 *sequences from 2720 species have been deposited in GenBank [10] as of 02 Feb 2009 compared to 13,347 *ef1α *sequences from 7353 species and 375,287 *COI *sequences from 46,385 species in BOLD [11]. This may, in part, be explained by alignment problems which have limited use of *ITS2 *in phylogenetic studies of more distantly related taxa. Advances in predicting the secondary structure of *ITS2 *enables alignment of *ITS2 *data from more distantly related taxa and increases its utility above the genus level [12,13]. In this paper we show that the inclusion of secondary structure information improves phylogeny estimation with *ITS2 *in a large radiation of blue butterflies and renders *ITS2 *a useful nuclear marker in phylogenetic studies. Furthermore, we suggest that *ITS2 *is a promising nuclear candidate for barcode studies, in addition to the mitochondrial marker *COI *.

The Lycaenidae are the second largest family of butterflies with about 6000 species worldwide. Among them is a large radiation of ca 130 Palaearctic species, i.e., the subgenus *Agrodiaetus *. It is extraordinary in Metazoa for its extreme interspecific variation of chromosome numbers, which is present even among closely related species that are often very similar or identical in phenotype [14-17]. Recently, the radiation has become the focus of several molecular phylogenetic studies in order to unravel the evolution of morphological and karyological characters [18-21] and to evaluate the barcoding approach [22]. All these studies employed *COI *as the main genetic marker. Wiemers [18] additionally used *ITS2 *as a secondary marker, but phylogenetic resolution without the inclusion of *COI *remained unsatisfactory, and the alignment had to be confined to the subtribe Polyommatina due to alignment problems. Kandul et al. [19] included *ef1α *as an additional nuclear marker in a small subset of taxa, but the marker hardly provided any phylogenetic signal and was therefore abandoned in subsequent studies [20,21]. Our aim is to compare and evaluate the phylogenetic trees based on *COI *with independent evidence from the nuclear *ITS2 *incorporating sequence, as well as, secondary structure information.

Without doubt, DNA sequence data are an extremely valuable source of information to infer phylogenetic relationships. Another usage of these data has recently come into the focus of both biological scientists and stakeholder groups and attracted much controversy among them: their usage to delimit and identify species [22-33]. Although *COI *has been the marker of choice for the barcoding campaign, *ITS2 *is a successful alternative. This is especially true in groups where *COI *fails to work well, e.g. in fungi [34], where it was used in combination with *ITS1 *, and, most recently, in diatoms [35]. Furthermore, it has been recently claimed that structural differences in *ITS2 *are predictive of species limits. In this view, pairings of CBCs (= compensatory base changes) provide an indication for sexual incompatibility [36], while their absence indicates intercrossing ability [37]. As the investigated taxonomic group provides an interesting and opportune example, a further aim of this study is to test, whether these claims also apply for the large and very recent radiation of the subgenus *Agrodiaetus *with an origin about 2.51-3.85 million years ago [19,21].

## Results

### Sequencing and alignment results

PCR products amplified successfully from all recently collected ethanol-preserved material, while dried material which had been successfully used for PCR of the mitochondrial cytochrome c oxidase I (*COI *) failed to consistently achieve successful PCR amplification of *ITS2 *. Furthermore, in 11% of sequencing reactions, incomplete sequences were obtained, probably caused by polymerase slippage at positions with highly repetitive motifs. Usually, it was still possible to obtain a complete sequence by sequencing from 5' and 3' ends such that the sequences only rarely remained incomplete after extended sequencing efforts. Incomplete sequences were excluded from the analysis as they may be result from co-amplified pseudogenes or not homogenized *ITS2 *copies. No obvious problems with intragenomic sequence variation were encountered in the remaining sequences -- all electropherograms obtained were readable over their entire length. Thus, we assume to have no problems associated with non-homogenized *ITS2 *copies, what has been reported in other *ITS *studies [38-41] and is discussed in several reviews [42,43]. Sequence length varied between 450 bp (in *Tarucus theophrastus *) and 602 bp (in *Allotinus portunus *and *Lysandra corydonius *). Sequence length variation in *Agrodiaetus *was between 530 bp (in *A. kurdistanicus *) and 563 bp (in *A. dama *). Nucleotide composition was typical for RNA with a slight overrepresentation of guanine (U : C : A : G = 0.234 : 0.261 : 0.203 : 0.302).

Alignment was successful for all sequences of the tribe Polyommatini (including six subtribes), as well as for the outgroup (Miletini: *Allotinus portunus *). Alignment difficulties were encountered with sequences of three other tribes (Theclini, Eumaeini and Lycaenini) which were therefore excluded from the analysis.

The alignment had 1024 positions of which 419 were variable and 235 were parsimony-informative (with gaps treated as missing data). Within *Agrodiaetus *, 131 positions were variable and 58 were parsimony informative.

### Phylogeny of *Polyommatus*

**Figure 1:**
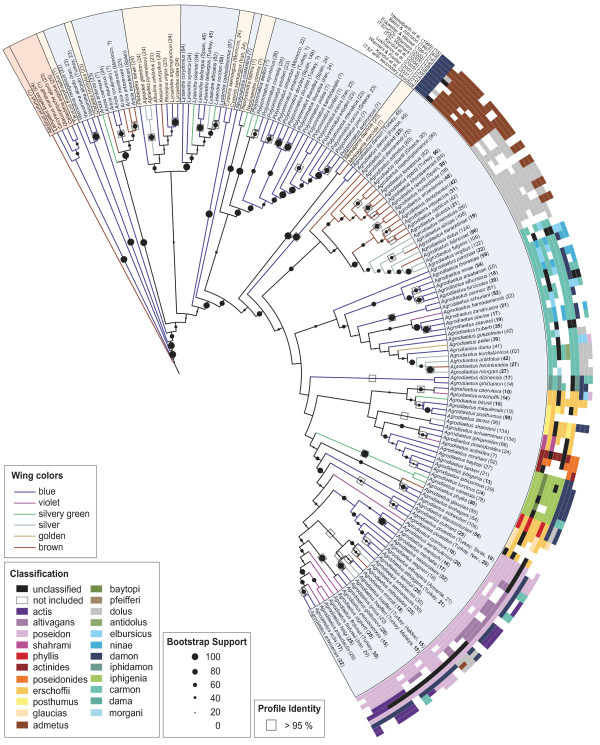
**Profile Neighbour-Joining (PNJ) tree of *ITS2***. *ITS2 *PNJ tree of 140 Lycaenidae species belonging to the tribe Polyommatini (Polyommatinae) and rooted with *Allotinus portunus *(Miletinae: Miletini) as outgroup. Bootstrap support values and profile identities > 95% are indicated on branches above nodes. Upperside wing colouration of males is indicated by branch colouration, using 6 different classes following Lukhtanov et al. (2005) [20]. Modal chromosome numbers are indicated in brackets after the species name (**bold **= gene sequence and karyotype data obtained from the same specimen; *italics *= sequence and karyotype data of a different individual from the same population [18-21]). Classification schemes of the present and other studies are coded by coloured rings around the tree. References to the corresponding studies are given in square brackets.

According to the Profile Neighbour Joining (= PNJ) tree (fig. 1), the genus *Polyommatus *represents a monophyletic unit with the exception of its subgenus *Lysandra *. The subgenus *Lysandra *is clearly monophyletic but its placement within *Plebejus *s.l. is unsupported. Some systematic treatments have united *Lysandra *with *Meleageria *, but the two subgenera appear distinctly distant from each other in our analysis.

The remaining subgenera (*Agrodiaetus, Meleageria, Polyommatus *s.str., *Neolysandra *) together form a monophyletic group with a bootstrap support of 88%. Regarding these subgenera, the monophyly of the subgenus *Agrodiaetus *is supported with a bootstrap value of 74%. The sister group to *Agrodiaetus *appears to be either the subgenus *Meleageria *or *Polyommatus *s.str. The latter subgenus includes taxa which have sometimes been placed in subgenera *Sublysandra *and *Plebicula *. While the taxa attributed to *Sublysandra *(*P. cornelia, P. aedon *and *P. myrrhinus *) appear to form a monophyletic cluster at the base of the remaining species of *Polyommatus *, the subgenus *Plebicula *(in which *P. dorylas, P. escheri, P. amandus *and *P. thersites *have sometimes been included) does not appear as a monophyletic entity. The taxa of the subgenus *Neolysandra *appear at a basal position relative to the other *Polyommatus *subgenera. The relationships of the remaining Polyommatina genera with each other and with *Polyommatus *are not well supported, except for the monophyly of *Aricia *. Nonetheless, the subtribe Polyommatina received high bootstrap support (95%) and the members of all other Lycaenidae tribes are positioned outside this cluster.

### Phylogeny of *Agrodiaetus*

*Agrodiaetus damon *(the two sequences from France and Turkey are identical) appears to be the sister taxon to all other *Agrodiaetus *. Unfortunately, the bootstrap support for this position is low. However, a single base-pair substitution is present at position 918 in the alignment that is a further support for the basal position of *A. damon *(although weak). At this position, all other *Agrodiaetus *sequences bear a guanine while *A. damon *and the remaining species of the genus *Polyommatus *bear an adenine base. The following major clades are supported by bootstrap values ≥ 50 among the remaining *Agrodiaetus *species as indicated in fig. 1 (bootstrap values in brackets): *admetus *clade (54%), *dolus *clade (81%), *carmon *clade (50%), *actinides *clade (62%), *iphigenia *clade (59%), *glaucias *clade (56%), *poseidon *clade (79%).

Additionally, there are some minor clades. Most of them are poorly supported and include only two species whose sequences are very similar or identical: *iphidamon *clade (13%, p-distance: 0.006), *erschoffii *clade (57%, p-distance: 0.011), *posthumus *clade (40%, p-distance: 0.002-0.006), *shahrami *clade (9%, p-distance: 0.000), *phyllis *clade (99%, p-distance: 0.000).

The remaining three species cluster with low bootstrap support: *A. valiabadi *as sister to the *admetus *and *dolus *clades (40%), *A. pierceae *as sister to the *carmon *clade (37%), and *A. klausschuriani *as sister to the *poseidon *clade (52%).

The phylogenetic relationships among the clades are usually poorly supported by bootstrap values with the exception of the *admetus *and *dolus *clades which form a clade together with *A. valiabadi *with a bootstrap support of 64%.

A classification based on *Agrodiaetus *clades with bootstrap support ≥ 50% is presented in fig. 1, together with classifications based on previous publications. A comparison of molecular based classifications reveals that 7 major clades are repeatedly found. Their support values are given in table 1.

**Table 1:** Support values for major clades in different analyses

Gene(s) & Reference	***ITS2***	***ITS2***	***COI***[18]	***ITS2***[18]	***COI+***	***ITS2***[18]	***COI+COII***[19]		***COI+COII ***[20]		***COI***[22]	***COI+COII ***[21]		
**Methods**	**PNJ**	**NJ**	BI*	BI*	**BI**	**MP**	**BI**	**MP**	**BI**	**ML**	NJ*	**ML**	**MP**	**BI**

*admetus*	54	45	100	84	100	100	100	100	100	100	98	100	100	100

*dolus*	*81*	*64*	100	100	100	100	100	100	100	100	90	100	100	100

*carmon*	50	0	0	81	100	100	100	73	100	88	9	88	74	100

*actinides*	62	42	53	<50	56	97	100	97	100	100	0	<50	<50	38

*iphigenia*	59	57	0	*91*	97	63	98	72	100	84	11	86	75	100

*erschoffii*	0	0	100	0	100	97	100	0	0	60	45	56	<50	<50

*poseidon*	79	0	100	*65*	100	98	100	96	100	96	63	97	97	100

Methods: **BI **= Bayesian inference, **ML **= Maximum Likelihood, **MP **= Maximum Parsimony, **NJ **= Neighbour-Joining, **PNJ **= Profile Neighbour Joining; *Support values taken from unpublished data

### Biogeographical patterns in *Agrodiaetus*

**Figure 2:**
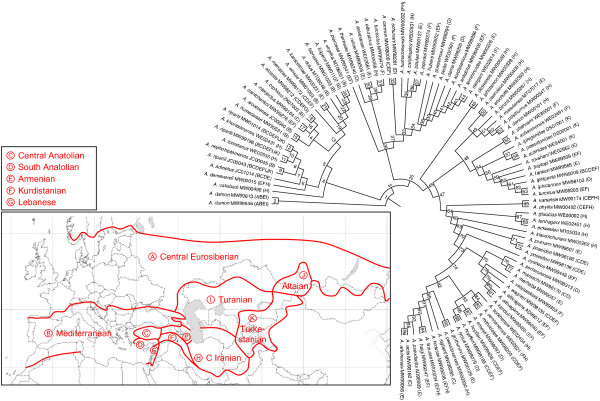
**PNJ tree of *ITS2 *and biogeographical regions**. *ITS2 *PNJ tree of 90 *Agrodiaetus *species and a map of biogeographical regions used for DIVA analysis. Occurrences in biogeographical regions are indicated by letters (A-K) after the species name and voucher code number according to the labels used in the map. Internal nodes in the tree are numbered consecutively.

According to the dispersal-vicariance model implemented in DIVA, the origin of *Agrodiaetus *remains uncertain, but the ancestral biogeographical areas of most major clades are quite precisely inferred (fig. 2, table 2 &3). An exception is the *admetus *clade whose ancestral area appears to encompass almost the entire range of the subgenus, with the exception of the Central Eurosiberian and Lebanese regions. The reason for this result, however, might be due to the poor taxonomy of this clade. It consists only of monomorphic species which hardly differ in phenotype and possess high chromosome numbers. The precise count of such high chromosome numbers is very difficult with standard karyological techniques [18]. Molecular results (of *ITS2 *as well as *COI *[18]) indicate that *A. ripartii *, the most widespread member of this clade, is not monophyletic and consists of several distinct species. The ancestral area of the closely related *dolus *clade also remains ambiguous but is confined either to the Mediterranean, the Central Anatolian, the Armenian, or Kurdistanian region. Most members of the *dolus *clade are also monomorphic or have high chromosome numbers. Therefore its taxonomy is contentious as well and this might have influenced the results. An illustrative example is given in the following section. The ancestral areas of the remaining clades appear to be restricted to four biogeographical regions. The Kurdistanian region is home to the *carmon *clade (as well as to the small Iranian *shahrami *clade) while the *iphigenia *and *poseidon *clades seem to have originated in the neighbouring Armenian region. (The latter clade might also have originated from both.) With the exception of the Turkestanian *actinides *clade, the remaining smaller clades (*erschoffii, posthumus, glaucias *) appear to have originated in the Central Iranian region.

**Table 2:** Distribution of *Agrodiaetus *species in biogeographical regions used for DIVA analysis

***Species***	Distribution												***Species***	Distribution											
*A. achaemenes*						F							*A. karacetinae*					E							
	
*A. actinides*											K		*A. khorasanensis*								H				
	
*A. actis*			C										*A. klausschuriani*								H				
	
*A. admetus*		B	C	D	E								*A. kurdistanicus*						F						
	
*A. ainsae*		B											*A. lorestanus*								H				
	
*A. alcestis*			C	D	E	F	G						*A. lycius*				D								
	
*A. altivagans*					E	F							*A. maraschi*			C	D								
	
*A. antidolus*					E	F							*A. masulensis*					E							
	
*A. arasbarani*					E								*A. menalcas*			C	D	E	F						
	
*A. aroaniensis*		B											*A. merhaba*					E							
	
*A. artvinensis*					E								*A. mithridates*			C	D	E	F						
	
*A. baytopi*					E	F							*A. morgani*						F						
	
*A. birunii*								H					*A. nephohiptamenos*		B										
	
*A. caeruleus*								H					*A. ninae*					E							
	
*A. carmon*			C		E	F							*A. orphicus*		B										
	
*A. cyaneus*					E	F							*A. paulae*					E							
	
*A. dama*				D									*A. peilei*						F						
	
*A. damon*	A	B			E				I				*A. phyllis*			C		E	F		H				
	
*A. dantchenkoi*					E	F							*A. pierceae*					E	F						
	
*A. darius*								H					*A. poseidon*			C	D	E							
	
*A. demavendi*					E	F		H					*A. poseidonides*											K	
	
*A. dizinensis*								H					*A. posthumus*								H				
	
*A. dolus*		B											*A. pseudactis*					E							
	
*A. eckweileri*								H					*A. pseudoxerxes*								H				
	
*A. elbursicus*								H					*A. putnami*					E							
	
*A. ernesti*				D									*A. ripartii*		B	C	D	E	F			I	J	K	
	
*A. erschoffii*								H					*A. rovshani*					E							
	
*A. fabressei*		B											*A. schuriani*				D								
	
*A. femininoides*					E								*A. sennanensis*						F		H				
	
*A. firdussii*					E	F		H					*A. sertavulensis*				D								
	
*A. fulgens*		B											*A. shahrami*						F						
	
*A. glaucias*								H					*A. sigberti*			C									
	
*A. gorbunovi*					E								*A. sorkhensis*								H				
	
*A. guezelmavi*				D									*A. tankeri*					E							
	
*A. haigi*					E	F							*A. tenhageni*								H				
	
*A. hamadanensis*						F		H					*A. theresiae*				D								
	
*A. hopfferi*			C	D	E	F							*A. turcicolus*						F						
	
*A. huberti*					E	F							*A. turcicus*					E	F						
	
*A. humedasae*		B											*A. valiabadi*								H				
	
*A. interjectus*			C										*A. vanensis*			C		E	F		H				
	
*A. iphicarmon*				D									*A. vaspurakani*						F						
	
*A. iphidamon*								H					*A. virgilius*		B										
	
*A. iphigenia*		B	C	D	E	F							*A. wagneri*			C	D	E	F						
	
*A. iphigenides*											K		*A. zapvadi*						F						
	
*A. kanduli*					E	F							*A. zarathustra*								H				

The abbreviations for the biogeographical regions are: A: Central Eurosiberian, B: Mediterranean, C: Central Anatolian, D: South Anatolian, E: Armenian, F: Kurdistanian, G: Lebanese, H: Central Iranian, I: Turanian, J: Altaian, K: Turkestanian

**Table 3:** Ancestral distributions according to DIVA analysis

Node	Regions included in alternative distributions											Alternative distributions
1	A	B	C	D	E	F		H	I	J	K	ABCDEFHIJK

2	A											A

3		B	C	D	E	F		H	I	J	K	BCDEFHIJK

4		B	C	D	E	F		H	I	J	K	BCDEHIJK, BCDEFHIJK

5		B	C	D	E	F		H	I	J	K	BCDEIJK, BCDEFIJK, BCDEHIJK, BCDEFHIJK

6		B	C	D	E	F		H	I	J	K	more than 10 distributions

7		B	C	D	E	F		H	I	J	K	more than 10 distributions

8		B	C	D	E	F		H	I	J	K	more than 10 distributions

9		B	C	D	E	F			I	J	K	more than 10 distributions

10		B	C	D	E	F		H	I	J	K	more than 10 distributions

11		B	C	D	E	F		H	I	J	K	more than 10 distributions

12		B	C	D	E	F		H	I	J	K	more than 10 distributions

13		B	C		E	F						B, C, E, F

14		B	C		E	F						B, BC, BE, BF

15		B										B

16		B	C	D	E	F	G					more than 10 distributions

17			C		E	F						CE, CF, CEF

18		B	C	D	E	F	G					more than 10 distributions

19		B	C	D	E	F						more than 10 distributions

20		B			E							B, BE

21		B			E							BE

22		B										B

23		B										B

24		B										B

25						F		H				FH

26						F						F

27						F						F

28				D	E	F		H				DF, DEF, DFH, DEFH

29					E	F		H				EF, EFH

30					E							E

31						F		H				FH

32						F						F

33			C	D	E	F						DF, CDF, DEF, CDEF

34						F						F

35						F						F

36					E	F		H				FH, EFH

37					E	F						EF

38						F						F

39				D		F						DF

40						F						F

41				D		F						DF

42						F						F

43						F						F

44					E	F						EF

45								H				H

46								H				H

47								H				H

48					E	F		H			K	EH, FH, EFH, HK, EHK, FHK, EFHK

49								H				H

50								H				H

51								H				H

52					E			H				EH

53								H				H

54					E	F					K	EF, FK, EFK

55						F						F

56					E						K	EK

57											K	K

58											K	K

59					E							E

60					E							E

61					E							E

62					E							E

63				D	E	F						DE, DEF

64								H				H

65								H				H

66								H				H

67								H				H

68								H				H

69					E	F		H				EH, FH, EFH

70					E	F						E, EF

71					E							E

72					E							E

73				D	E	F						E, DE, F, EF, DEF

74				D	E	F		H				D, E, DE, F, EF, DEF, DEH, EFH, DEFH

75			C	D	E	F						E, DE, DF, EF, CEF, DEF, CDEF

76			C	D	E							D, DE, CDE

77			C	D	E							CE, DE, CDE

78					E	F						F, EF

79					E	F						E, F

80					E	F						E, F

81					E	F						E, F

82				D	E	F		H				E, DE, EF, DEF, EH, DEH, EFH, DEFH

83			C	D	E	F		H				more than 10 distributions

84			C	D	E	F		H				more than 10 distributions

85			C	D	E	F		H				more than 10 distributions

86				D								D

87				D								D

88				D								D

89					E							E

90					E			H				EH

91			C		E							CE

92					E							E

93					E							E

94					E							E

95					E							E

96			C		E							CE

The abbreviations for the biogeographical regions are: A: Central Eurosiberian, B: Mediterranean, C: Central Anatolian, D: South Anatolian, E: Armenian, F: Kurdistanian, G: Lebanese, H: Central Iranian, I: Turanian, J: Altaian, K: Turkestanian

### Compensatory base changes (CBCs) in *Agrodiaetus*

A maximum of only 3 CBCs are found among the 140 investigated species-level taxa of Lycaenidae. One of them occurs between members of the *Agrodiaetus *+*Polyommatus *+*Meleageria *clade and the remaining Lycaenidae species (with the exception of *Neolysandra fatima *). In 64% of pairwise species comparisons (and even 99.8% of congeneric comparisons) no CBCs are found. Within *Agrodiaetus *hardly any species is distinguished by a CBC, but some major clades can be delimited by hemi-CBCs such as the *iphigenia *and *dolus *clade. Due to the low number of CBCs and hemi-CBCs, the NJ trees created from CBC or hemi-CBC distance matrices provide little resolution (data not shown).

Although CBCs are uncommon within Polyommatini, most species differ in their *ITS2 *sequence. Identical haplotypes were only found in very few sets of taxa (table 4). Most of them concern taxa with questionable species status [18,44]. For example, *A. karacetinae *differs only in karyotype and COI sequence from *A. alcestis *, but not in any morphological characters ("karyospecies"). Its position in fig. 1 (as sister to *A. ainsae *) is an artefact caused by a single missing nucleotide at position 628 in the alignment which causes a change in secondary structure making it similar to *A. ainsae *. The sequence of the latter taxon is most similar to that of *A. fulgens *, and its distant position to this species in fig. 1 can also be explained by several missing nucleotides. According to recent karyological research, *A. ainsae *appears to be conspecific with *A. fulgens *and the name *A. ainsae *was therefore synonymised with *A. fulgens *[45].

**Table 4:** List of identical *ITS2 *haplotypes in different taxa

*Aricia artaxerxes/A. montensis *(Spain)
*Lysandra albicans/L. coridon*

*Polyommatus eroides/P. menelaos*

*Polyommatus icarus *(Greece)/*P. andronicus*

*Agrodiaetus ripartii *(Greece)/*A. nephohiptamenos*

*Agrodiaetus alcestis/A. karacetinae*

*Agrodiaetus femininoides/A. morgani*

*Agrodiaetus shahrami/A. achaemenes*

*Agrodiaetus tankeri/A. iphigenia*

*Agrodiaetus altivagans *(Armenia)/*A. kanduli*

*Agrodiaetus firdussii *(Iran)/*A. haigi/A. actis/A. artvinensis*

## Discussion

### Secondary structure information improves phylogenetic signal in *ITS2*

Wiemers [18] used a mostly comparable set of taxa for phylogenetic inference from *ITS2 *but did not include secondary structure information. Although most major clades recovered in our analysis were also found in the Bayesian analysis by Wiemers [18], none of our major clades were recovered with bootstrap support values ≥ 50% in the Maximum Parsimony (MP) analysis of Wiemers [18]. The *poseidon *clade was also not recovered in the Bayesian 80% consensus tree presented. (This clade - with the exclusion of *A. putnami *- only received a Bayesian support of 0.65, Wiemers unpubl., table 1). In a Neighbour Joining (NJ) analysis calculated without secondary structure information only two of the major clades recovered in the PNJ analysis received bootstrap values ≥ 50% while two clades received lower bootstrap values and the remaining two were not recovered at all (table 1). Thus, in a direct comparison of two NJ algorithms (with vs. without secondary structure, table 1), secondary structure information apparently amplifies the phylogenetic information in the data set. Further improvement in phylogeny estimation is to be expected if secondary structure information can be incorporated in Maximum Likelihood (ML) or Bayesian inference (BI) methods, because these character-based methods can be superior compared to distance based methods which discard character-state information.

One disadvantage of using secondary structure information appears to be its sensitivity to missing data in stem regions. Even small amounts of missing data can cause artefacts in phylogeny estimation of closely related taxa with very similar sequences (viz. *A. alcestis *and *A. karacetinae *).

### Phylogenetic signal of *ITS2 *is comparable to *COI *in *Agrodiaetus*

In agreement with *COI *analyses [18], *ITS2 *data support the monophyly of Polyommatina which includes the genera *Chilades, Plebejus *and *Polyommatus *. The monophyly of the genera *Plebejus *and *Polyommatus *, however, is not fully supported. This is due to the placement of the subgenus *Lysandra *within *Plebejus *, which however has no bootstrap support and is probably caused by long-branch attraction. Such a placement is also in conflict with the Bayesian analysis of *COI *which places *Lysandra *within the genus *Polyommatus *[18]. The *ITS2 *sequences of subgenus *Lysandra *are peculiar in having several longer inserts with repetitive motifs, e.g. in position 70-133 in the alignment. It is noteworthy, on the one hand, that none of the analyses supports a sister-relationship between *Lysandra *and *Meleageria *, even though members of these genera can hybridize with each other [46-48] and therefore were considered to be very closely related [15]. On the other hand, *Cyaniris *is found within *Plebejus *in the *COI *tree but basal within *Polyommatus *in the *ITS2 *tree, both times with low support values. Here, the *COI *analysis appears to be more affected by long-branch attraction.

Within *Agrodiaetus *, the phylogenetic analysis of *ITS2 *recovers clades which are mostly congruent to those obtained from an analysis of *COI *+ *COII *(= cytochrome c oxidase II). Of particular interest is the confirmation of the sister relationship between *A. damon *and the remaining *Agrodiaetus *species that was not or only very weakly supported in the *COI *analyses. *ITS2 *and *COI *also agree in the monophyly and sister relationship of the *admetus *and *dolus *clades, only the position of *A. valiabadi *differs (within the *dolus *clade in *COI *, but sister to *admetus *+*dolus *in *ITS2 *). The *carmon *clade is also recovered in the *COI *+*COII *analyses but includes the *iphidamon *clade in the analyses by Lukhtanov et al. [20] and Kandul et al. (2007) [21]. Kandul et al. (2004) [19] split this group into three clades although one of them (clade VII) only appears in the MP analysis and has no bootstrap support. In the *COI *analyses by Wiemers [18] and Wiemers & Fiedler [22], which are based on shorter sequences, the *carmon *group receives no bootstrap support. Similarly, the *iphigenia *clade is only recovered in the mtDNA analyses based on the long 1969 bp section of *COI *+*COII *. The *poseidon *clade is recovered in the *COI *analyses, as well. Kandul et al. [19] split this clade into three subclades but the addition of further taxa revealed that they are not monophyletic and thus should be combined [20,21]. Most interesting is the *actinides *clade in the *ITS2 *tree which suggests a close relationship between *A. actinides, A. poseidonides *and *A. iphigenides *. Although previous analyses have also suggested a close relationship among these taxa, it was never well supported. The relationships of the remaining clades (*glaucias, erschoffii, posthumus, shahrami, phyllis *) are not well supported in the *ITS2 *tree. Previous analyses using *COI *[18-20] have suggested a close relationship of these clades, but their combination into an inclusive *erschoffii *clade was only very weakly supported by the latest *COI *analysis [21], probably due to the inclusion of additional taxa (such as *A. eckweileri *). The only major discrepancy is the placement of *A. klausschuriani *in the *ITS2 *analyses (sister to the *poseidon *clade) compared to the *COI *analyses (within the *erschoffii *clade), but both placements are only very weakly supported. The missing support for the relationships between the major clades also applies to the *COI *analyses. Most analyses, however, agree in the basal position of the *admetus *+*dolus *clade and all of them recover the *poseidon *clade at the tip of the tree.

We conclude that the phylogenetic signal of *ITS2 *is comparable to the signal of a much longer fragment of *COI */*COII *. This is surprising since the rate of parsimony-informative characters is lower in *ITS2 *than in *COI *[18]. Apparently these characters are, however, less "noisy" than those of *COI *, which are almost completely confined to 3^rd ^codon positions.

### *ITS2 *confirms weaknesses of morphological classifications

**Figure 3:**
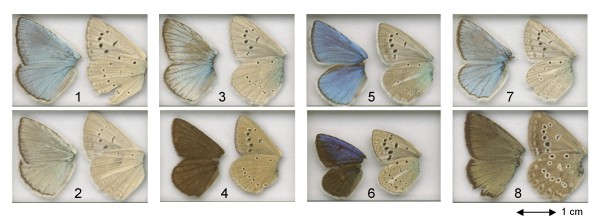
**Male wing vouchers of sister species pairs with different upperside colouration**. 1-2: *Agrodiaetus lycius *(MW98079) - *A. hopfferi *(MW98189). 3-4: *Agrodiaetus fulgens *(MW01107) - *A. fabressei *(MW01039). 5-6: *Agrodiaetus caeruleus *(MW00409) - *A. erschoffii *(MW00393). 7-8: *Meleageria daphnis *(MW98029) - *M. marcida *(MW00290). Uppersides are shown on the left and undersides on the right side of each image

Fig. 1 reveals little congruence between previous classifications based on morphological characters [14,15,49] and those on molecular data (*COI *or *ITS2 *). The main reason for this is the small number of available morphological characters (mostly slight differences in wing colouration) which are highly susceptible to homoplasy. Illustrative examples are morphology-based groupings formed by species with discoloured males, in which the iridescent bluish colouration on the wing upperside is replaced by a brown, golden or silvery colour (the *admetus *and *dolus *groups). Discolouration of males is coupled with an expansion of the androconial patches, apparently due to a switch from a visual to a scent-based mate recognition system [18]. Although the molecular analyses also recover a clade containing exclusively discoloured males (the clade formed by the *admetus *and *dolus *sister-clades), the molecular data reveal that single discoloured species or small groups of them are also found in most other clades. Discoloured species also appear in many other subgenera of *Polyommatus *and related genera which usually have bluish males. In the sister species pair, *M. daphnis/M. marcida *, the discolouration of the latter taxon (which possibly represents only a conspecific population of the former) is probably an adaptation to the specific climatic conditions (low solar radiation) on the north side of Elburs mountains [50]. Such sister species pairs with differing male upperside colouration are also found in *Agrodiaetus *, e.g. *A. fabressei/fulgens, A. shahrami/achaemenes, A. erschoffii/caeruleus *and *A. hopfferi/lycius *(fig. 3).

In some butterfly groups with similar wing patterns, genitalia provide important features for identification and classification. Unfortunately, they are very similar in all *Agrodiaetus *species, possess only few usable characters and therefore have only rarely been evaluated. The little available evidence, however, appears to be more congruent with molecular data than with wing pattern characters. Coutsis [51] analyzed the genitalia of several *Agrodiaetus *taxa which had previously been regarded as subspecies of *Agrodiaetus iphigenia *due to their similar wing colouration, among them *A. iphidamon *and *A. iphigenides *. He concluded that genitalia differences rule out conspecifity. According to the molecular results these taxa belong to different clades. *A. iphidamon *and *A. dizinensis *have been placed in different groups according to wing pattern characters [49], but they share a synapomorphic character in their genitalia: the shape of the labides is short, pointed and "dagger-like" (Coutsis, pers. comm.). Molecular results also clearly show that they are closely related. The monomorphic *Agrodiaetus *species of the *admetus *and *dolus *clades differ in karyotype but are difficult or impossible to identify based on wing pattern characters. Members of these two clades, however, differ in the length of their valves relative to their body size, those in the *admetus *clade (with the possible exception of *A. admetus *) being shorter than those in the *dolus *clade [52-54]. A comprehensive treatment of the genitalia of *Polyommatina *is currently in preparation (Coutsis, pers. comm.).

### Historical biogeography

The results of our DIVA analysis confirm earlier assumptions (e.g. [18]) that Eastern Anatolia, Transcaucasia and Iran are the main centres of *Agrodiaetus *radiation. Although the origin of the subgenus could not be inferred with this method, the ancestral biogeographical areas of most major clades are placed in this region. Most interestingly, the origin of each of these clades seems to be confined to a single region (or possibly two neighbouring regions in one case). These results support the evolutionary significance of the clades obtained from the molecular analyses (*ITS2 *as well as *COI/COII *).

### CBCs as predictors of sexual incompatibility and the utility of *ITS2 *to delimit species

Due to the low number of CBCs (and hemi-CBCs) in Lycaenidae, these structural markers cannot be used to predict species limits in the family. Although this does not preclude the possibility that a CBC is a sufficient condition to distinguish species [36], an absence of CBCs cannot be used to predict intercrossing ability as suggested by Coleman [37].

This deficiency does not mean that *ITS2 *sequences cannot be used to delimit species. Even in the young radiation of *Agrodiaetus *, scarcely any two species have identical *ITS2 *haplotypes, while the same haplotype may be found in distant populations of the same species, e.g. *Agrodiaetus damon *from France and Turkey. On the other hand, sequence differences among populations and among individuals in a single population do exist [18], and we currently lack sufficient intraspecific *ITS2 *sequence data to check for the existence of a barcode gap or diagnostic DNA characters [22,25]. Available intraspecific *ITS2 *sequences usually cluster together in the PNJ tree. Exceptions occur in species complexes with disputable species borders (*A. ripartii *and *A. altivagans *) and in *Polyommatus icarus *: the Iranian *P. icarus *sequence does not cluster with conspecific sequences but with the almost identical sequence of *P. forsteri *, and is even identical with that of an Iranian specimen (voucher code ILL071) of *Polyommatus icadius *[44]. The latter is a Central Asian species, whose phenotype is very similar to *P. icarus *, but which is well differentiated in *ITS2 *and was only recently discovered in Iran [44]. The phenotype of the Iranian *P. icarus *specimen, however, is typical for *P. icarus *and its *COI *sequence is almost identical to those of *P. icarus *from Greece and Anatolia, where *P. icadius *does not occur [22]. Therefore it is possible that the specimen (MW00412) actually represents a hybrid between *P. icarus *and *P. icadius *. Some evidence for introgressive hybridization between these two taxa comes from the Altai where *P. icarus *and *P. icadius *share identical *COI *haplotypes [55]. Although this complex needs further research it is an example for the importance of analysing a fast nuclear locus in addition to the mitochondrial *COI *.

## Conclusions

Our analyses show that *ITS2 *can be a suitable phylogenetic marker not only for closely related groups of species, but also for higher taxa. In the family Lycaenidae, secondary structure information enabled the alignment of sequences from different subtribes of the tribe Polyommatini.

In *Agrodiaetus*, six major clades were obtained which are corroborated by independent evidence from mitochondrial DNA, genitalia structure, as well as our biogeographical analysis. These clades, however, do not correspond with traditional classifications, which were mainly based on the very limited set of wing pattern characters.

The use of secondary structure information with Profile Neighbour Joining also increased resolution and bootstrap support in the subgenus *Agrodiaetus *to the extent that *ITS2 *phylogenetic trees provide a resolution comparable to *COI *.

In insects, *ITS2 *currently appears to be the only available and well tested nuclear DNA marker which is informative enough to resolve the phylogeny of young radiations such as *Agrodiaetus *. Therefore we recommend the use of this marker as an addition to mitochondrial markers (like *COI *) in order to prevent erroneous estimation of species trees caused by introgressive hybridization, incomplete lineage sorting or horizontal gene transfer. Although introgression of mitochondrial DNA (mtDNA) appears to be less common in Lepidoptera than in most other Metazoa due to their female-heterogametic sex chromosome system [56] and Haldane's rule [57], recent work shows that such cases exist (Wiemers unpublished; [58]) and therefore should not be ignored.

We cannot, however, corroborate the use of CBCs to delimit species, because CBCs are very rare even among distantly related species in Lycaenidae and, at least, for this group their absence is not a useful predictor for sexual compatibility as claimed by Coleman et al. [37].

## Methods

### Material

A total of 156 Lycaenidae *ITS2 *sequences were included for our analysis. Of these, 17 were exclusively determined for this study. The remainders were selected from the phylogenetic analysis of the PhD thesis by the first author [18]. Five of these sequences were improved in quality by repeating the sequencing procedure.

Generally, only one sequence per species was retained, except for taxa with a large range or with considerable geographic variation. In the latter case, two sequences representing this variation were retained. Selection criterion was the sequence quality in order to minimize ambiguities. For three species, the only available sequence was of insufficient quality and therefore these taxa were excluded from the analysis (*Agrodiaetus surakovi, Aricia eumedon, Plebejides pylaon *).

Most sequences belong to *Agrodiaetus *(97), the others to closely related genera of the same subtribe Polyommatina (54) or other subtribes within the tribe Polyommatini (5 sequences). *Allotinus portunus *(Miletinae) was chosen as outgroup because it was the only non-Polyommatini sequence available within Lycaenidae which could successfully be aligned. Alignment of sequences from the tribes Lycaenini, Theclini and Eumaeini failed, despite the fact that they are held to be more closely related to Polyommatini according to the morphology-based classification by Eliot [59].

All sequences have been deposited in GenBank [10] with LinkOuts provided to images of the voucher specimens deposited with MorphBank [60] (table 5). Annotation changes of existing entries after HMM-Annotation were as well submitted to this database. No further complete *ITS2 *sequences of Lycaenidae are currently available from GenBank. The voucher specimens and DNA extractions are currently stored by the first author at the Department of Animal Biodiversity, Vienna University, but will eventually be deposited at the Alexander Koenig Research Institute and Museum of Zoology in Bonn (Germany).

**Table 5:** List of taxa included in this study, their provenance and accession numbers

Species	Country	Locality	Collecting Date	Voucher code	Morph-Bank id	GenBank Accession
*Agriades glandon*	Italy	Stilfser Joch (2300 m), Bozen-Südtirol	27.07.2008	MW08069		GQ166180

*Agriades pyrenaicus*	Turkey	Çaglayan (1500 m), Erzincan	05.07.1999	MW99018	65226	AY556659

*Agrodiaetus achaemenes*	Iran	Gardaneh ye Cheri, W Samsami (2800-3000 m), Bakhtiari	21.07.2002	WE02491		AY556740

*Agrodiaetus actinides*	Kirgizia	Aram-Kungei valley, Alytyn Dara river (3000 m), West Transalai	11.07.1994	WE94001		AY556753

*Agrodiaetus actis*	Turkey	Gökpinar (1700 m), Sivas	25.07.1998	MW98162	65049	AY556633

*Agrodiaetus admetus*	Greece	Mt. Taiyetos (1200-1300 m), Peloponnisos	14.06.2001	JC01014	64205	AY556733

*Agrodiaetus ainsae*	Spain	Sta. Maria (500 m), Huesca	20.07.2001	MW01053	64811	AY556610

*Agrodiaetus alcestis*	Turkey	Saimbeyli falls (1500 m), Adana	28.07.1998	MW98212	65098	AY556641

*Agrodiaetus altivagans*	Armenia	Gnyshik village (1800-2200 m), Transcaucasia	20.07.1998	AD98012	64133	AY556717

*Agrodiaetus altivagans*	Turkey	Güzeldere Geç. (2500 m), Van	17.07.1999	MW99240	65448	AY556676

*Agrodiaetus antidolus*	Turkey	Dez Çay (1500 m), Hakkari	22.07.1999	MW99406	65614	AY556692

*Agrodiaetus arasbarani*	Iran	Mahmutabad, W Kaleybar (2200-2400 m), Azarbayjan-e Sharqi	29.07.2002	WE02661		AY556747

*Agrodiaetus aroaniensis*	Greece	Mt. Helmos (1350 m), Peloponnisos	04.07.2000	JC00040	64181	AY556725

*Agrodiaetus artvinensis*	Turkey	Kiliçkaya (1350 m), Artvin	08.07.1999	MW99058	65266	AY556663

*Agrodiaetus baytopi*	Turkey	Çatak (2000-2200 m), Van	18.07.1999	MW99309	65517	AY556684

*Agrodiaetus birunii*	Iran	Veresk (1800-1950 m), Mazandaran	18.07.2000	MW00267	64474	AY556578

*Agrodiaetus caeruleus*	Iran	Hajiabad (2150 m), Golestan	23.07.2000	MW00409	64616	AY556589

*Agrodiaetus carmon*	Turkey	Karabayir (1400 m), Antalya	11.07.1998	MW98009	64896	AY556622

*Agrodiaetus cyaneus*	Turkey	Zernek Brj. (1900 m), Van	23.07.1999	MW99448	65656	AY556696

*Agrodiaetus dama*	Turkey	Gündüzbey (1300 m), Malatya	27.07.1998	MW98205		AY556640

*Agrodiaetus damon*	Turkey	Köskköy (1900 m), Erzurum	28.07.1999	MW99546	65753	AY556705

*Agrodiaetus damon*	France	Col de Tende (1850 m), Alpes Maritimes	17.08.1999	MW99613	65820	AY556714

*Agrodiaetus dantchenkoi*	Turkey	Kurubaş Geçidi (2200 m), Van	17.07.1999	MW99276	65484	AY556679

*Agrodiaetus darius*	Iran	Dizin Pass (3000 m), Tehran	12.07.2000	MW00101	64310	AY556560

*Agrodiaetus demavendi*	Iran	Samqabad (1900-2100 m), Tehran	09.07.2000	MW00015	64224	AY556552

*Agrodiaetus dizinensis*	Iran	Dizin Pass (3200-3300 m), Tehran	04.08.2000	MW00539	64746	AY556599

*Agrodiaetus dolus*	France	Auriol, La Roussargue (550 m), Bouches-du-Rhône	19.07.2006	MT06048		GQ166173

*Agrodiaetus eckweileri*	Iran	Fenjan, Surian (3000 m), Fars	08.07.2005	MT05034		GQ166172

*Agrodiaetus elbursicus*	Iran	Pul-e Zanguleh (2400 m), Mazandaran	11.07.2000	MW00058	64267	AY556556

*Agrodiaetus ernesti*	Turkey	Dedegöl Geçidi (1700 m), Isparta	21.07.1998	MW98097	64984	AY556626

*Agrodiaetus erschoffii*	Iran	Hajiabad (2150 m), Golestan	23.07.2000	MW00393	64600	AY556588

*Agrodiaetus fabressei*	Spain	Abejar (1100 m), Soria	19.07.2001	MW01039	64797	AY556608

*Agrodiaetus femininoides*	Iran	Qazayd Dagh (2300 m), Zanjan	16.07.2000	MW00226	64435	AY556573

*Agrodiaetus firdussii*	Iran	Qazayd Dagh (2300 m), Zanjan	16.07.2000	MW00234	64443	AY556576

*Agrodiaetus firdussii*	Turkey	Çaglayan (1500 m), Erzincan	05.07.1999	MW99006	65214	AY556655

*Agrodiaetus fulgens*	Spain	Sta. Coloma de Queralt (700 m), Tarragona	23.07.2001	MW01107	64856	AY556615

*Agrodiaetus glaucias*	Iran	Voluyeh (1500-1600 m), Mazandaran	24.05.2000	WE00002	65829	AY556736

*Agrodiaetus gorbunovi*	Iran	Ahar Pass (1800-1850 m), Azarbayjan-e Sharqi	13.07.2000	MW00129	64338	AY556565

*Agrodiaetus guezelmavi*	Turkey	Taşkent (1450 m), Konya	04.08.1998	MW98294	65180	AY556651

*Agrodiaetus haigi*	Turkey	Güzeldere Geç. (2500 m), Van	17.07.1999	MW99247	65455	AY556677

*Agrodiaetus hamadanensis*	Iran	Safedabad (2000 m), Tehran	10.07.2000	MW00032	64241	AY556554

*Agrodiaetus hopfferi*	Turkey	Gündüzbey (1300 m), Malatya	27.07.1998	MW98189	65076	AY556638

*Agrodiaetus hopfferi*	Turkey	Dez Çay (1500 m), Hakkari	22.07.1999	MW99408	65616	AY556694

*Agrodiaetus huberti*	Turkey	Kop Geçidi (2350 m), Bayburt	29.07.1999	MW99552	65759	AY556707

*Agrodiaetus humedasae*	Italy	Pondel (900 m), Aosta	14.08.1999	MW99591	65798	AY556710

*Agrodiaetus interjectus*	Turkey	Çiftlik (1900 m), Erzurum	14.07.1999	MW99164	65372	AY556671

*Agrodiaetus iphicarmon*	Turkey	Dedegöl Geçidi (1700 m), Isparta	21.07.1998	MW98103	64990	AY556627

*Agrodiaetus iphidamon*	Iran	Shakuh (2600 m), Golestan	21.07.2000	MW00328	64535	AY556584

*Agrodiaetus iphigenia*	Turkey	Çaglayan (1500 m), Erzincan	05.07.1999	MW99009	65217	AY556656

*Agrodiaetus iphigenides*	Uzbekistan	Kitabsky national reserve (1500-2500 m)	08.06.2001	DS01001	64175	AY556722

*Agrodiaetus kanduli*	Turkey	Çatak (1600-1900 m), Van	24.07.1999	MW99465	65673	AY556697

*Agrodiaetus karacetinae*	Iran	Qazayd Dagh (2300 m), Zanjan	16.07.2000	MW00231	64440	AY556574

*Agrodiaetus khorasanensis*	Iran	5 km SW Firizi (1700-1900 m), Khorasan	16.07.2002	WE02431		AY556737

*Agrodiaetus klausschuriani*	Iran	Veresk (1800-1950 m), Mazandaran	18.07.2000	MW00262	64471	AY556577

*Agrodiaetus kurdistanicus*	Turkey	Çatak (1600-1900 m), Van	18.07.1999	MW99286	65494	AY556680

*Agrodiaetus lorestanus*	Iran	30 km W Dorud (2100 m), Lorestan	25.07.2002	WE02535	65837	AY556743

*Agrodiaetus lycius*	Turkey	Cukurelma (1300 m), Antalya	15.07.1998	MW98079	64966	AY556625

*Agrodiaetus maraschi*	Turkey	Gökpinar (1700 m), Sivas	25.07.1998	MW98170	65057	AY556634

*Agrodiaetus masulensis*	Iran	Rudbar S Janat (2600-3000 m), Mazandaran	03.07.2007	MT07017		GQ166175

*Agrodiaetus menalcas*	Turkey	Gökpinar (1700 m), Sivas	25.07.1998	MW98172	65059	AY556635

*Agrodiaetus merhaba*	Turkey	Kiliçkaya (1350 m), Artvin	08.07.1999	MW99057	65265	AY556662

*Agrodiaetus mithridates*	Turkey	Gündüzbey (1300 m), Malatya	27.07.1998	MW98203	65090	AY556639

*Agrodiaetus morgani*	Iran	40 km SW Saqqez (1800-1900 m), Kordestan	27.07.2002	WE02614		AY556745

*Agrodiaetus nephohiptamenos*	Greece	Mt. Orvilos (1200-2100 m), Macedonia	07.07.2000	JC00045	64186	AY556728

*Agrodiaetus ninae*	Turkey	Ağrı (1800 m), Ağrı	26.07.1999	MW99508	65716	AY556701

*Agrodiaetus orphicus*	Bulgaria	Stara Planina Mts., Karandila Nature Park (1000 m), Sliven	29.07.2007	ZK07003		GQ166185

*Agrodiaetus paulae*	Iran	Ahar Pass (1800-1850 m), Azarbayjan-e Sharqi	13.07.2000	MW00127	64336	AY556564

*Agrodiaetus peilei*	Iran	Qamchiyan, 30 km N Chenareh (1800-1900 m), Kordestan	27.07.2002	WE02591	65839	AY556744

*Agrodiaetus phyllis*	Iran	Polur (2200 m), Tehran	26.07.2000	MW00452	64659	AY556592

*Agrodiaetus pierceae*	Turkey	Güzeldere Geç. (2600 m), Van	19.07.1999	MW99341	65549	AY556686

*Agrodiaetus poseidon*	Turkey	Zelve (1100 m), Nevşehir	22.07.1998	MW98138	65025	AY556630

*Agrodiaetus poseidon*	Turkey	Gökpinar (1700 m), Sivas	25.07.1998	MW98180	65067	AY556636

*Agrodiaetus poseidonides*	Tajikistan	Safedou (2500 m), Darvaz Mts.	23.06.2000	DS00001	65845	AY556721

*Agrodiaetus posthumus*	Iran	Shakuh (2600 m), Golestan	21.07.2000	MW00347	64554	AY556586

*Agrodiaetus pseudactis*	Armenia	Gnyshik village (1800-2200 m), Transcaucasia	20.07.1998	AD98009	64130	AY556716

*Agrodiaetus pseudoxerxes*	Iran	Shakuh (2600 m), Golestan	21.07.2000	MW00330	64537	AY556585

*Agrodiaetus putnami*	Turkey	Ağrı (1800 m), Ağrı	26.07.1999	MW99501	65709	AY556700

*Agrodiaetus ripartii*	Greece	Mt. Helmos (1350-1500 m), Peloponnisos	21.06.2000	JC00043	64184	AY556727

*Agrodiaetus ripartii*	Spain	Ubierna (900 m), Burgos	18.07.2001	MW01014	64773	AY556603

*Agrodiaetus ripartii*	Turkey	Çaglayan (1500 m), Erzincan	15.07.1999	MW99196	65404	AY556673

*Agrodiaetus rovshani*	Iran	Mahmutabad, W Kaleybar (2200-2400 m), Azarbayjan-e Sharqi	29.07.2002	WE02662		AY556748

*Agrodiaetus schuriani*	Turkey	Gezbeli Geçidi (1800 m), Kayseri	30.07.1998	MW98261	65147	AY556646

*Agrodiaetus sennanensis*	Iran	20 km E Mahabad (1900 m), Azarbayjan-e Gharbi	28.07.2002	WE02621		AY556746

*Agrodiaetus sertavulensis*	Turkey	Yellibeli Geçidi (1800 m), Karaman	06.08.1998	MW98313	65199	AY556652

*Agrodiaetus shahrami*	Iran	30 km N Chelgerd Pass (3000-3200 m), Bakhtiari	23.07.2002	WE85001		AY556752

*Agrodiaetus sigberti*	Turkey	Ala Daglar (2700 m), Kayseri	31.07.1998	MW98285	65171	AY556650

*Agrodiaetus sorkhensis*	Iran	Kuh-e-Sorkh, Kadkan (2100-2500 m), Khorasan	17.07.2002	WE02454	65833	AY556739

*Agrodiaetus tankeri*	Turkey	Kop Geçidi (2350 m), Bayburt	29.07.1999	MW99565	65772	AY556709

*Agrodiaetus tenhageni*	Iran	Kuh-e-Sorkh, Kadkan (2100-2500 m), Khorasan	17.07.2002	WE02451	65831	AY556738

*Agrodiaetus theresiae*	Turkey	Saimbeyli falls (1200-1500 m), Adana	29.07.1998	MW98240	65126	AY556645

*Agrodiaetus turcicolus*	Turkey	Erek Dagi (2200 m), Van	25.07.1999	MW99479	65687	AY556699

*Agrodiaetus turcicus*	Turkey	Çaglayan (1500 m), Erzincan	15.07.1999	MW99203	65411	AY556674

*Agrodiaetus valiabadi*	Iran	5 km S Valiabad (1900 m), Mazandaran	30.07.2000	MW00498	64705	AY556594

*Agrodiaetus vanensis*	Turkey	Çaglayan (1500 m), Erzincan	15.07.1999	MW99174	65382	AY556672

*Agrodiaetus vaspurakani*	Turkey	Güzeldere Geç. (2500 m), Van	19.07.1999	MW99353	65561	AY556687

*Agrodiaetus virgilius*	Italy	Assergi, Gran Sasso (1000 m), Abruzzo	20.07.2006	MT06051		GQ166174

*Agrodiaetus wagneri*	Turkey	Zelve (1100 m), Nevşehir	22.07.1998	MW98136	65023	AY556629

*Agrodiaetus zapvadi*	Turkey	Zernek Brj. (1900 m), Van	20.07.1999	MW99374	65582	AY556689

*Agrodiaetus zarathustra*	Iran	30 km W Dorud (2100 m), Lorestan	25.07.2002	WE02531	65834	AY556741

*Albulina orbitulus*	Austria	Mitteralm, Grossglockner (1600 m), Salzburg	04.07.2006	MW06120		GQ166176

*Allotinus portunus*	Indonesia	Ujung Kulon National Park (0 m), West Java	27.01.2008	MW08003		GQ166177

*Aricia anteros*	Turkey	Erciyes Dagi (2000 m), Kayseri	30.07.1998	MW98270	65156	AY556648

*Aricia artaxerxes*	Greece	Mt. Taiyetos (1180-1200 m), Peloponnisos	16.06.2000	JC00055	64193	AY556730

*Aricia cramera*	Spain	Sta. Maria (500 m), Huesca	20.07.2001	MW01061	64819	AY556612

*Aricia isauricus*	Turkey	Kagizman (1400 m), Kars	11.07.1999	MW99097	65305	AY556666

*Aricia montensis*	Spain	Abejar (1100 m), Soria	19.07.2001	MW01048	64806	AY556609

*Aricia montensis*	Morocco	Oukaimeden (2700 m), Marrakech	15.07.2002	MW02033	64883	AY556620

*Aricia torulensis*	Turkey	Torul (1100 m), Gümüshane	04.07.1999	MW99001	65209	AY556654

*Cacyreus marshalli*	France	Maruéjols-les-Gardons (100 m), Hérault	27.07.2001	MW01120	64864	AY556543

*Celastrina argiolus*	Morocco	Oukaimeden (2300 m), Marrakech	09.07.2002	MW02008	64872	AY556547

*Chilades trochylus*	Turkey	Dez Çay (1500 m), Hakkari	22.07.1999	MW99425	65633	GQ166186

*Cyaniris semiargus*	Iran	Takht-e Suleyman (3500-3700 m), Mazandaran	01.08.2000	MW00525	64732	AY556597

*Cyaniris semiargus*	Morocco	Oukaimeden (2700 m), Marrakech	15.07.2002	MW02034	64884	AY556621

*Glaucopsyche alexis*	Turkey	Cukurelma (1300 m), Antalya	13.06.2006	MK06007		GQ166171

*Kretania eurypilus*	Turkey	Çatak (1600-1900 m), Van	18.07.1999	MW99303	65511	AY556683

*Lampides boeticus*	Morocco	Tourchte (1400 m), Marrakech	14.07.2002	MW02028	64880	AY556546

*Lycaeides argyrognomon*	Austria	Wien-Donaustadt (200 m)	19.06.2008	MW08032		GQ166178

*Lycaeides idas*	Italy	Burgeis (1800-1900 m), Bozen-Südtirol	26.07.2008	MW08065		GQ166179

*Lysandra albicans*	Spain	Boltana (650 m), Huesca	22.07.2001	MW01092	64842	AY556614

*Lysandra bellargus*	Spain	Ilarduya (550 m), Alava	17.07.2001	MW01011	64770	AY556602

*Lysandra bellargus*	Turkey	Dez Çay (1500 m), Hakkari	23.07.1999	MW99446	65654	GQ166183

*Lysandra caelestissimus*	Spain	Moscardon (1600 m), Teruel	30.07.1996	OK96022	65826	AY556735

*Lysandra coridon*	Italy	Pondel (900 m), Aosta	14.08.1999	MW99612	65819	AY556713

*Lysandra corydonius*	Turkey	Gaziler (1800 m), Iğdır	26.07.1999	MW99514	65722	AY556702

*Lysandra ossmar*	Turkey	Zelve (1100 m), Nevşehir	22.07.1998	MW98155	65042	GQ166181

*Lysandra syriaca*	Turkey	Saimbeyli falls (1500 m), Adana	28.07.1998	MW98228	65114	AY556643

*Meleageria daphnis*	Turkey	Gülübeli Geçidi (1500 m), Fethiye	12.07.1998	MW98029	64916	AY556623

*Meleageria marcida*	Iran	Veresk (1800-1950 m), Mazandaran	18.07.2000	MW00290	64497	AY556580

*Neolysandra coelestina*	Turkey	Çaglayan (1500 m), Erzincan	05.07.1999	MW99013	65221	AY556657

*Neolysandra corona*	Iran	Takht-e Suleyman (3000 m), Mazandaran	31.07.2000	MW00504	64711	AY556595

*Neolysandra fatima*	Turkey	Çatak (1600-1900 m), Van	18.07.1999	MW99301	65509	AY556682

*Plebejidea loewii*	Turkey	Saimbeyli falls (1500 m), Adana	28.07.1998	MW98220	65106	AY556642

*Plebejus argus*	Iran	Shemshak (2900 m), Tehran	12.07.2000	MW00116	64325	AY556563

*Polyommatus aedon*	Iran	Shakuh (2600 m), Golestan	21.07.2000	MW00326	64533	AY556583

*Polyommatus amandus*	Morocco	Oukaimeden (2300 m), Marrakech	09.07.2002	MW02001	64865	AY556617

*Polyommatus amandus*	Turkey	Köskköy (1900 m), Erzurum	07.07.1999	MW99047	65255	AY556661

*Polyommatus andronicus*	Greece	Mt. Falakro (1650 m), Macedonia	09.07.2000	JC00061	64197	AY556731

*Polyommatus celina*	Morocco	Oukaimeden (2300 m), Marrakech	09.07.2002	MW02006	64870	AY556618

*Polyommatus cornelia*	Turkey	Gezbeli Geçidi (1800 m), Kayseri	30.07.1998	MW98264	65150	AY556647

*Polyommatus dorylas*	Spain	Ubierna (900 m), Burgos	18.07.2001	MW01019	64778	AY556605

*Polyommatus dorylas*	Turkey	Çaglayan (1500 m), Erzincan	05.07.1999	MW99014	65222	AY556658

*Polyommatus eroides*	Greece	Rodopi Mts. (1200 m), Macedonia	08.07.2000	JC00042	64183	AY556726

*Polyommatus escheri*	Greece	Mt. Falakro (1650 m), Macedonia	09.07.2000	JC00039	64180	AY556724

*Polyommatus forsteri*	Iran	Takht-e Suleyman (3500-3700 m), Mazandaran	01.08.2000	MW00530	64737	AY556598

*Polyommatus icarus*	Greece	Mt. Falakro (1650 m), Macedonia	09.07.2000	JC00063	64199	AY556732

*Polyommatus icarus*	Iran	Hajiabad (2150 m), Golestan	23.07.2000	MW00412	64619	AY556590

*Polyommatus juno*	Israel	Mt. Hermon (2050 m)	05.07.2008	DB08003		GQ166170

*Polyommatus kamtshadalis*	Russia	Sokol, Magadan, NE Siberia	10.07.2002	RU02003		GQ166184

*Polyommatus menelaos*	Greece	Mt. Taiyetos (1180-1200 m), Peloponnisos	16.06.2000	JC00029	64178	AY556723

*Polyommatus myrrhinus*	Turkey	Kop Geçidi (2200 m), Erzurum	29.07.1999	MW99550	65757	AY556706

*Polyommatus thersites*	Iran	Veresk (1800-1950 m), Mazandaran	18.07.2000	MW00302	64509	AY556581

*Polyommatus thersites*	Spain	Triste (600 m), Huesca	21.07.2001	MW01083	64835	AY556613

*Tarucus theophrastus*	Morocco	Tourchte (1400 m), Marrakech	14.07.2002	MW02025	64877	AY556619

*Vacciniina alcedo*	Iran	Samqabad (1900-2100 m), Tehran	09.07.2000	MW00024	64233	AY556553

*Vacciniina alcedo*	Turkey	Dez Çay (1500 m), Hakkari	22.07.1999	MW99430	65638	GQ166182

*Vacciniina morgianus*	Iran	Takht-e Suleyman (3600 m), Mazandaran	31.07.2000	MW00517	64724	AY556596

Follow this link (http://www.morphbank.net/Browse/BySpecimen/) to search Morph Bank numbers mentioned in column 6.

In many *Agrodiaetus *species groups, especially among the monomorphic, i.e., "brown" species, karyotypes are important for species identification. Therefore in most of the specimens included in molecular analysis, the karyotypes were studied [18] using squash techniques [61,62].

Upperside wing colouration of males was classified according to the method of Lukhtanov et al. (2005) [20]. One additional colour class ("golden" for golden brown) was added for *Agrodiaetus peilei *, a species which was not assessed in their study.

### Taxonomy

The subgenera of *Polyommatus *and *Plebejus *have often been attributed generic rank in recent literature, and we follow this convention for the purposes of the present paper. The following subgenera are included in these genera: *Polyommatus *: *Cyaniris, Polyommatus, Meleageria, Lysandra, Neolysandra, Agrodiaetus *; *Plebejus *: *Plebejus, Plebejidea, Plebejides, Lycaeides, Kretania, Albulina, Agriades, Aricia, Vacciniina *. The subgeneric treatment follows Hesselbarth et al. [15] with the following two exceptions: *Lysandra *(synonymised with *Meleageria *by Hesselbarth et al. [15]) and *Lycaeides *(synonymised with *Plebejus *by Hesselbarth et al. [15]).

The status of many taxa in the genus *Polyommatus *is questionable, especially in the subgenus *Agrodiaetus *which includes many recently described species, some based on disputable evidence. Taxonomic revisions and further research are needed to clarify the status of these taxa. At present, we have retained most species in order to facilitate comparisons with published studies, although some have been synonymised recently. For example, *Agrodiaetus ainsae *has been synonymised with *A. fulgens *[45] and Vodolazsky et al. [44] treat several *Polyommatus *taxa as subspecies or synonyms of *P. eros *(*P. kamtshadalis, P. eroides *and *P. menelaos *) and *P. icarus *(*P. andronicus *and *P. juno *).

### Laboratory protocols

DNA was extracted from thorax tissue recently collected and preserved in 100% ethanol using QIAGEN^® ^DNeasy Tissue Kit according to the manufacturer's protocol for mouse tail tissue. Occasionally, only dried material was available and either thorax or legs were used for DNA extraction. Amplification of DNA was conducted using the polymerase chain reaction (PCR). The reaction mixture (for a total reaction volume of 25 μl) included: 1 μl DNA, 16.8 μl ddH20, 2.5 μl 10 × PCR II buffer, 3.2 μl 25 mM MgCl2, 0.5 μl 2 mM dNTP-Mix, 0.25 μl Taq Polymerase and 0.375 μl 20 pm of each primer. The two primers used were ITS3 (5'-GCA TCG ATG AAG AAC GCA GC-3') and ITS4 (5'-TCC TCC GCT TAT TGA TAT GC-3') [63].

PCR was conducted on thermal cyclers from BIOMETRA^® ^(models UNO II or T-GRADIENT) or ABI BIOSYSTEMS^® ^(model GENEAMP^® ^PCR-System 2700) using the following profiles: initial 4 minutes denaturation at 94°C and 35 cycles of 30 seconds denaturation at 94°C, 30 seconds annealing at 55°C and 1 minute extension at 72°C.

PCR products were purified using purification kits from PROMEGA^® ^or SIGMA^® ^and checked with agarose gel electrophoresis before and after purification.

Cycle sequencing was carried out on BIOMETRA^® ^T-GRADIENT or ABI BIOSYSTEMS^® ^GENEAMP^® ^PCR-System 2700 thermal cyclers using sequencing kits of MWG BIOTECH^® ^(for LI-COR^® ^automated sequencer) or ABI BIOSYSTEMS^® ^(for ABI^® ^377 automated sequencer) according to the manufacturers' protocols and with the following cycling times: initial 2 minutes denaturation at 95°C and 35 cycles of 15 seconds denaturation at 95°C, 15 seconds annealing at 49°C and 15 seconds extension at 70°C. Primers used were the same as for the PCR reactions for the ABI (primer 1 for forward and primer 2 for independent reverse sequencing). Electrophoresis of sequencing reaction products was carried out on LI-COR^® ^or ABI^® ^377 automated sequencers using the manufacturer's protocols. Electropherograms were edited and aligned using the LaserGene^® ^Software SeqMan Pro Version 7.1.0 by DNASTAR^®^.

### Data analysis

#### Secondary Structure Prediction

**Figure 4:**
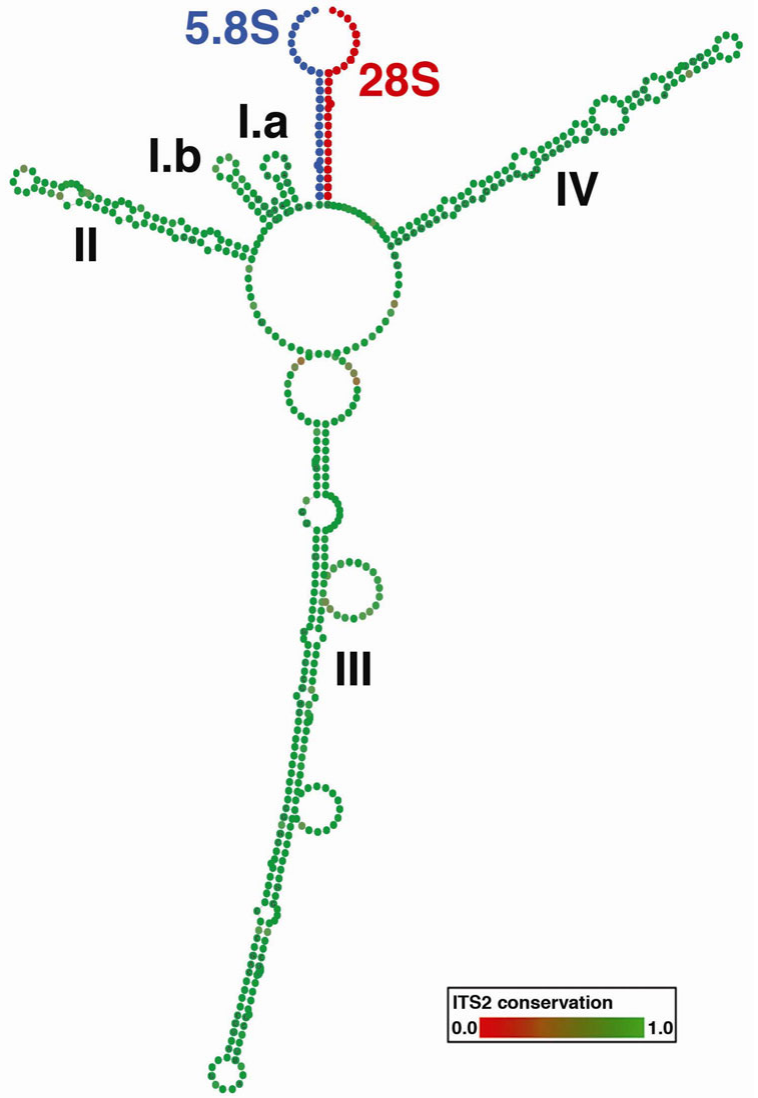
**Conserved ITS2 secondary structure of the Polyommatina**. The proximal stem of hybridized 5.8S (blue) and 28S (red) rDNA is included. Helices are numbered in Roman numerals. Two small helices are found near the beginning, which are referred to as helices I.a and I.b. The first (basal) internal bulge of helix II with two nucleotides mismatching one nucleotide is the typical U-U mismatch found in the second helix of ITS2 structures throughout the Eukaryota. Degree of conservation is displayed in colour grades from green (conserved) to red (unconserved). The complete structure represents the 51% consensus of aligned structures without gaps.

**Figure 5:**
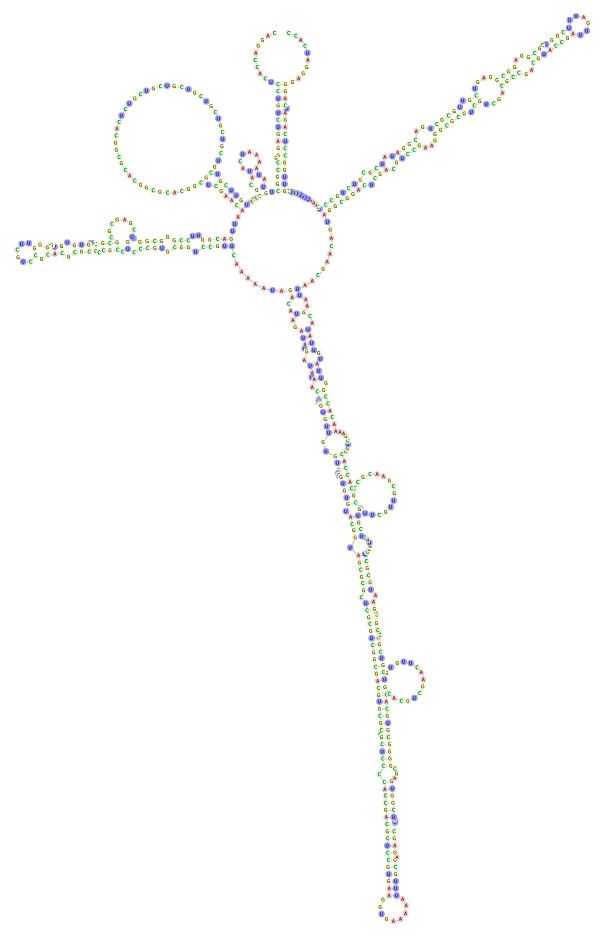
**ITS2 secondary structure of *Lysandra syriaca***. In the distal loop of helix I.b an insertion of nucleotides is present in the genus *Lysandra *. Based on homology modelling with a template in which these nucleotides are absent (*Neolysandra *), the nucleotide insertions remain unpaired. This is a distinctive feature for the genus.

Data analysis followed the method described in Schultz & Wolf [64] for secondary structure phylogenetics. All retained *ITS2 *sequences were delimited and cropped with the HMM-based annotation tool present at the *ITS2 *database ([65]; E-value < 0.001, metazoan HMMs). This tool furthermore integrates a visual check for the 5.8S/28S hybridization as the ITS2 proximal stem. Incorrect folding of this region is a good indication for pseudogenes [66]. All sequences of this study passed this test with a correct folding, so that we are confident to exclude pseudogenes in this study. Furthermore, according to Álvarez & Wendel [42], *ITS *pseudogenes have lowered secondary structure stability and an increase in AT content via deaminations. This was not the case for our complete *ITS2 *sequences, since their secondary structures were stable and the GC content of each sequence was clearly above 50%. The proximal stem (25 nucleotides of *5.8S *as well as *28S *rDNA) was included to preserve a conserved margin of the alignment. For several sequences, nucleotides near the 3' end of the proximal stem were ambiguous. For these, nucleotides with more than 95% consensus within the remaining aligned sequences were adopted by the majority rule to preserve the marginal secondary structure of the RNA. The secondary structure of the *ITS2 *of *Neolysandra coelestina *(MW99013) was predicted with RNA structure 4.6 [67] and ported to Vienna format with CBCanalyzer 1.0.3 [68] (fig. 4). The structures of the remaining sequences were predicted by custom homology modelling at the *ITS2 *database [69-72] with the aforementioned structure as a template and at least 70% helix transfer (identity matrix, gap costs: gap open 15, gap extension 2). We further applied a Nussinov Algorithm (perl script) to each sequence to close additional base-pairs within helices, which were left open by homology modelling. For this procedure, no existing base pairs were removed, no pseudo-knots were allowed and exclusively Watson-Crick pairs were added (see fig. 5 for examples).

### Alignment and Phylogenetic Analyses

Sequences and secondary structures were automatically and synchronously aligned with 4SALE 1.5 [73,74]. 4SALE translates sequence-structure tuple information prior to alignment into pseudo-proteins. Pseudo-proteins were coded such that each of the four nucleotides may be present in three different states: unpaired, opening base-pair and closing base-pair. Thus, an *ITS2 *specific 12 × 12~scoring matrix was used for calculation of the alignment [73,74]. Sequence-structure alignment is available at the *ITS2 *database supplements page [75].

To determine evolutionary distances between organisms simultaneously on sequences and secondary structures we used Profile Neighbour Joining (PNJ) [76] as implemented in ProfDistS 0.98 [77,78]. The tree reconstructing algorithm works similar to the alignment method on a 12 letter alphabet comprised of the 4 nucleotides in three structural states (unpaired, paired left, paired right). We applied an *ITS2 *-specific general time reversible substitution model [73]. Profiles were automatically built for nodes with bootstrap support values (1000 replicates) above 70% or with at least 95% nucleotide identities. A profile is regarded as a sequence, however it is composed of probability distribution vectors instead of characters. PNJ is iterated until no more profiles can be defined according to our settings. The resulting tree was displayed with iTol v1.3.1[79] and further refined with CorelDRAW X3 (Corel Corporation, Ottawa, Canada). We utilized CBCanalyzer 1.1 [73,74] to detect CBCs and hemi-CBCs between sequence-structure pairs and to calculate a CBC tree. We used MEGA 4.0.1 [80] to calculate a matrix of p-distances and TCS 1.21 [81] to detect identical haplotypes. MEGA was also used to calculate the bootstrap support values (1000 replicates) of the NJ tree without secondary structure information using the Tamura-Nei model of nucleotide substitution with heterogeneous pattern among lineages and gamma distributed rates among sites. The appropriate model and the gamma parameter (0.8365) were calculated with MODELTEST 3.7 [82].

### Classification procedures

To evaluate the results of our approach we constructed a classification of *Agrodiaetus *based on major clusters with bootstrap values ≥ 50% and compared this classification with those constructed in similar ways from published studies which either used the same marker but without secondary structure information or the mitochondrial marker *COI *or both. The clusters were named after the taxonomically most senior taxon. Classifications from published studies were constructed in the following way:

• A classification for *ITS2 *without secondary structure information was constructed using major clusters from the Bayesian analysis conducted by Wiemers [18] with 84 *Agrodiaetus *species. Only groups with Bayesian posterior probabilities ≥ 0.80 were considered.

• From an analysis of 1969 bp *COI *and *COII *sequences from 55 *Agrodiaetus *species, Kandul et al. [19] proposed a classification of 12 major clades using Maximum Parsimony and Bayesian inference most of which have high bootstrap and Bayesian support. One notable exception is clade VII (*carmon *clade) which has no support and should have been combined with clade VI (*antidolus *clade) and clade VIII (*ninae *clade).

• Lukhtanov et al. [20] used an extended set of *COI *+*COII *sequences from 80 *Agrodiaetus *species and proposed 8 major clades based on Maximum Likelihood inference of phylogeny all of which are supported by bootstrap values > 50%.

• Kandul et al. [21] produced a Maximum Likelihood tree of a further extended set of *COI *+*COII *sequences from 105 *Agrodiaetus *taxa but did not provide a classification. We inferred one using major clades with support values MP ≥ 50%, ML ≥ 50% or BI ≥ 0.80.

• Wiemers & Fiedler [22] carried out a NJ analysis using a combination of *COI *sequences taken from Wiemers [18] and Lukhtanov et al. [20] which included a total of 116 *Agrodiaetus *species. Major clusters with bootstrap values ≥ 50% were used for the classification.

• A combined analysis of *ITS2 *and *COI *sequences of similar length (690 bp) from 88 *Agrodiaetus *species was carried out by Wiemers [18]. He proposed a classification based on clusters obtained with Bayesian inference using a support threshold for posterior probabilities of 0.95.

### Biogeographical analysis

A dispersal-vicariance analysis was conducted with the programme DIVA 1.2 [83] to infer the ancestral distributions in the phylogeny of *Agrodiaetus *. Since outgroup relationships of *Agrodiaetus *were not well resolved in previous studies, *A. damon *was used as the outgroup to the remaining *Agrodiaetus *species according to our complete PNJ analysis (Fig. 1). The distribution area of *Agrodiaetus *was divided into 11 biogeographical regions which are based on floral biogeographical regions [84]:

• C Eurosiberian: the Central European region (incl. the Central Siberian subregion) and the Pontic - South Siberian region

• Mediterranean: the Submediterranean and Mediterranean regions excl. the South Anatolian and Palestinian - Lebanese provinces

• C Anatolian: the Central Anatolian province in the Oriental Turanian region

• S Anatolian: the South Anatolian province in the Mediterranean region

• Armenian: the Armenian - NW Iranian province in the Oriental Turanian region

• Kurdistanian: the Kurdistanian - SW Iranian province in the Oriental Turanian region

• Lebanese: the Palestinian - Lebanese province in the Mediterranean region

• C Iranian: the Central Iranian, Hyrcanian, Turkmenian, and Balutchistanian provinces in the Oriental Turanian region

• Turanian: the Turanian subregion in the Oriental Turanian region

• Altaian: the Altaian region

• Turkestanian: the Turkestanian subregion in the Oriental Turanian region

Information on the occurrence of *Agrodiaetus *species in these regions was gathered from published distribution maps and regional faunistic monographs [15,85-95].

FigTree v.1.2.3 [96] was used to draw the tree with labelled internal nodes.

## Authors' contributions

The first author conceived and coordinated the study, performed most of the sampling and molecular genetic studies, analyzed data and drafted the manuscript. AK performed secondary structure predictions, alignment calculations and phylogenetic reconstructions under supervision of MWo. All authors read and approved the final manuscript.
